# Non-Traditional Cardiovascular Risk Factors: Tailored Assessment and Clinical Implications

**DOI:** 10.3390/jcdd12050171

**Published:** 2025-04-28

**Authors:** Francesco Perone, Marco Bernardi, Luigi Spadafora, Matteo Betti, Stefano Cacciatore, Francesco Saia, Federica Fogacci, Vikash Jaiswal, Elad Asher, Francesco Paneni, Salvatore De Rosa, Maciej Banach, Giuseppe Biondi Zoccai, Pierre Sabouret

**Affiliations:** 1 Cardiac Rehabilitation Unit, Rehabilitation Clinic “Villa delle Magnolie”, 81020 Castel Morrone, Caserta, Italy; 2 Department of Medical-Surgical Sciences and Biotechnologies, Sapienza University of Rome, 04100 Latina, Italy; marcobernardi23@gmail.com (M.B.); luigispadafora167@gmail.com (L.S.); gbiondizoccai@gmail.com (G.B.Z.); 3 Department of Clinical Sciences and Community Health, Cardiovascular Section, University of Milan, 20122 Milan, Italy; matteobetti1@gmail.com; 4 Department of Geriatrics, Orthopedics and Rheumatology, Università Cattolica del Sacro Cuore, L.go F. Vito 1, 00168 Rome, Italy; stefano.cacciatore01@icatt.it; 5 Fondazione Policlinico Universitario “Agostino Gemelli” IRCCS, L.go A. Gemelli 8, 00168 Rome, Italy; 6 Interventional Cardiology, IRCCS Azienda Ospedaliero-Universitaria di Bologna, Policlinico Sant’Orsola, 40138 Bologna, Italy; francescosaia@hotmail.com; 7 Hypertension and Cardiovascular Risk Research Center, Medical and Surgical Sciences Department, University of Bologna, 40138 Bologna, Italy; federicafogacci@gmail.com; 8 Department of Cardiovascular Research, Larkin Community Hospital, South Miami, FL 33431, USA; vikash29jaxy@gmail.com; 9 Jesselson Integrated Heart Center, Shaare Zedek Medical Center Jerusalem and Faculty of Medicine, Hebrew University of Jerusalem, Jerusalem 9103102, Israel; easher@szmc.org.il; 10 Center for Translational and Experimental Cardiology (CTEC), Deapartment of Cardiology, University Hospital Zürich and University of Zürich, Wagistrasse 12, Schlieren, 8952 Zurich, Switzerland; francesco.paneni@usz.ch; 11 University Heart Center, University Hospital Zurich, Ramistrasse 100, 8091 Zurich, Switzerland; 12 Department of Medical and Surgical Sciences, Magna Graecia University, 88100 Catanzaro, Italy; saderosa@unicz.it; 13 Ciccarone Center for the Prevention of Cardiovascular Disease, Division of Cardiology, Department of Medicine, Johns Hopkins University School of Medicine, 600 N. Wolfe St., Carnegie 591, Baltimore, MD 21287, USA; maciejbanach77@gmail.com; 14 Department of Preventive Cardiology and Lipidology, Medical University of Lodz (MUL), Rzgowska 281/289, 93-338 Lodz, Poland; 15 Maria Cecilia Hospital, GVM Care & Research, 48033 Cotignola, Italy; 16 Heart Institute and Action Group, Pitié-Salpétrière, Sorbonne University, 75013 Paris, France; cardiology.sabouret@gmail.com

**Keywords:** non-traditional risk factors, risk assessment, cardiovascular disease, traditional risk factors, cardiovascular prevention

## Abstract

Non-traditional cardiovascular risk factors (RFs) are increasingly emerging as important modifiers of cardiovascular risk (CVR), offering insights beyond traditional metrics like hypertension, diabetes, and dyslipidemia. These include novel biomarkers, chronic conditions (e.g., chronic kidney disease and chronic obstructive pulmonary disease), environmental exposures, chronic inflammation, infections, psychosocial factors, and sex-specific conditions, all of which influence the prediction, management, and outcomes of cardiovascular disease (CVD). These additional RFs may impact on CVD prediction and add valid information during tailored patient assessment and management. Therefore, a careful assessment of both traditional and non-traditional cardiovascular RFs, with a personalized treatment, could dramatically reduce the total CVD burden. Nevertheless, further research is needed to precisely estimate the magnitude of their impact as risk and prognosis modifiers in order to be included in future risk charts. This review provides a critical analysis of non-traditional RFs, their pathophysiological mechanisms, and their implications for personalized care. Integrating these factors into CVR assessment can reclassify patient risk categories, optimize therapeutic strategies, and improve prognosis. However, further research is needed to refine their inclusion in risk charts and evaluate their impact on public health outcomes. A tailored, multidisciplinary approach is essential to reduce the burden of CVD and associated mortality.

## 1. Introduction

**Figure 1:**
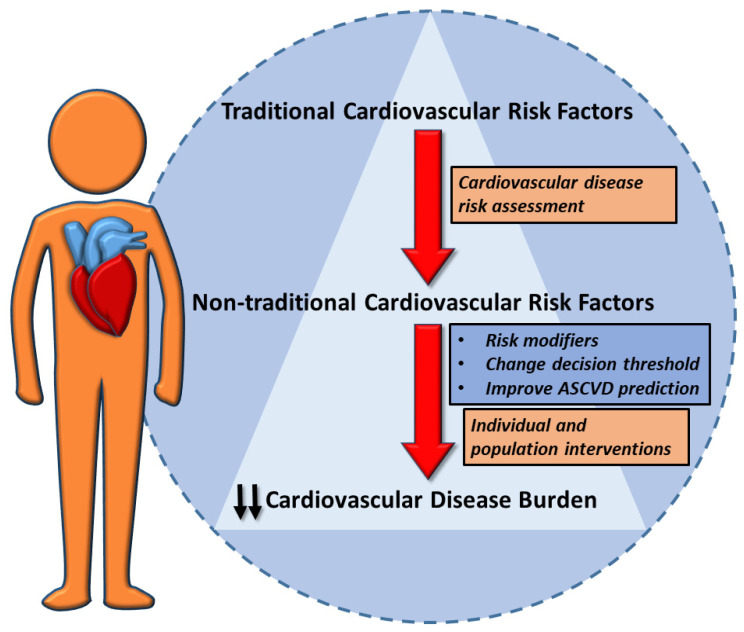
Role and impact of traditional and non-traditional cardiovascular risk factors during risk assessment.

Cardiovascular disease (CVD) represents the leading cause of morbidity and mortality worldwide, driven largely by well-established risk factors such as high blood pressure (HBP), diabetes, and dyslipidemia [1,2]. However, several studies highlight the role of non-traditional risk factors that contribute to CVD pathogenesis beyond these conventional metrics. These include biomarkers such as high-sensitivity C-reactive protein (hs-CRP), lipoprotein(a), and homocysteine, which reflect systemic inflammation and other atherogenic processes [3]. Conditions such as chronic kidney disease (CKD), cancers and their treatments, chronic obstructive pulmonary disease (COPD), and environmental exposures, particularly to air pollutants and extreme weather events, further increase cardiovascular risk by fostering oxidative stress, endothelial dysfunction, and vascular calcification [4]. CVD risk estimation is the first and key step for the tailored definition of risk category and treatment goals. A stepwise approach is necessary to prevent and treat atherosclerotic CVD risk factors [5]. Both traditional and non-traditional risk factors should be considered during CVD assessment to better define cardiovascular risk in order to provide personalized interventions to reduce CVD burden [6]. Indeed, in combination with conventional CVD risk factors, non-traditional risk factors could modify the cardiovascular risk evaluation, change the decision threshold, and improve the atherosclerotic CVD prediction (Figure 1, Table 1). This review critically examines the role of non-traditional risk factors in cardiovascular risk assessment, emphasizing their integration into clinical practice and their potential to inform personalized therapeutic strategies. By addressing these underexplored factors, we aim to enhance cardiovascular prevention and improve patient outcomes.

## 2. New Biomarkers

In cardiovascular risk assessment, new risk factors and/or biomarkers such as lipoprotein(a), hs-CRP, and homocysteine are increasingly recognized for their role in providing insights beyond traditional risk factors. These biomarkers reflect inflammatory, metabolic, and genetic mechanisms that contribute to CVD. Lipoprotein(a), commonly referred to as Lp(a), is a genetically determined lipoprotein variant that resembles low-density lipoprotein (LDL) but includes an additional protein, apolipoprotein(a), linked to apolipoprotein B-100 via a disulfide bond [7,8]. This unique structure contributes not only to atherogenic properties by promoting endothelial adhesion and foam cell formation but also to prothrombotic effects and oxidative modification of LDL particles. Elevated Lp(a) levels are associated with an increased risk of myocardial infarction (MI), stroke, and peripheral artery disease (PAD) due to their pro-inflammatory and pro-thrombotic effects [9]. Unlike LDL, lifestyle modifications and standard lipid-lowering treatments (LLTs) only have a marginal impact on Lp(a) concentrations. Newer LLTs, such as proprotein convertase subtilisin/kexin type 9 (PCSK9) inhibitors, decrease by 20% the Lp(a) levels which is not sufficient to decrease atherosclerotic events, whereas specific antisense oligonucleotides provide promising biological results by reducing Lp(a) levels by 80% from baseline, with ongoing phase III randomized clinical trials (RCTs) to assess their clinical efficacy [7,10]. European guidelines suggest (Class IIa, level B) measuring Lp(a) at least once in life, especially in subjects with personal or familial premature cardiovascular ischemic events [5]. Lp(a) measurement is particularly useful in cases where elevated low-density lipoprotein cholesterol (LDL-C) is detected despite high-intensity statin therapy. This phenomenon can be attributed to the fact that Lp(a) particles contain a cholesterol component that contributes to the total LDL-C measurement when calculated via equations or similar methods. Since Lp(a) cholesterol accounts for approximately 20–30% of the Lp(a) particle mass, individuals with high Lp(a) levels may present with persistently elevated LDL-C values, even when achieving optimal adherence to lipid-lowering therapy. This discrepancy underscores the importance of directly measuring Lp(a) in patients with unexpectedly high LDL-C despite aggressive statin treatment [11]. Furthermore, Lp(a) has been increasingly recognized as a key player also in the pathogenesis of aortic stenosis. Indeed, elevated Lp(a) levels are associated with accelerated valvular calcification and disease progression, independent of traditional cardiovascular risk factors. The pro-atherogenic and pro-inflammatory properties of Lp(a) contribute to valvular interstitial cell activation, oxidative stress, and osteogenic differentiation, promoting progressive calcification of the aortic valve [12].

Lp(a) plasma concentration is largely determined by genetic factors, with minimal influence from lifestyle or conventional lipid-lowering therapies. There is no clear evidence on Lp(a) risk thresholds. Recent EAS consensus suggests using ‘grey zones’ to rule-out or rule in cardiovascular risk. In practice, 30–50 mg/dL (or 75–125 nmol/L) indicate higher risk, while levels below 30 mg/dL (or 75 nmol/L) suggest lower risk [13].

Regarding hs-CRP, a marker of systemic inflammation correlated with the development and progression of atherosclerotic plaques and subsequent major adverse cardiovascular events (MACE). Elevated hs-CRP levels contribute to endothelial dysfunction, plaque instability, and an increased likelihood of acute coronary syndromes (ACSs), particularly in individuals with additional risk factors [14]. Epidemiological and interventional studies have demonstrated that elevated hsCRP levels (typically >2 mg/L) are associated with an increased risk of major adverse cardiovascular events (MACEs), even in patients receiving high-intensity lipid-lowering therapy [15]. Beyond its role in risk stratification, hs-CRP is also valuable as a contributor of residual risk [16,17]. Recent advancements in point-of-care hs-CRP testing allow for rapid assessment, making it feasible to monitor inflammatory responses and adjust treatment plans as needed, even in resource-limited settings. Further research is mandatory to confirm the utility of hs-CRP in clinical practice for timely adjustments in patient care based on inflammation levels [18]. Recent evidence suggests that targeting low-grade inflammation may be an effective strategy for reducing cardiovascular risk. Colchicine, an anti-inflammatory agent traditionally used for gout and pericarditis, has gained attention in cardiovascular prevention due to its ability to inhibit the NLRP3 inflammasome, thereby reducing interleukin-1β and interleukin-6 levels, which play a central role in atherogenesis and plaque instability. The LoDoCo2 trial demonstrated that in patients with chronic coronary disease, colchicine 0.5 mg daily significantly reduced the incidence of major adverse cardiovascular events (MACEs), including myocardial infarction and stroke, independent of lipid-lowering and antithrombotic therapy [19].

Instead, regarding homocysteine, it is an amino acid produced during the metabolism of methionine, and elevated levels are linked to endothelial damage, oxidative stress, and a pro-thrombotic state [20,21]. Hyperhomocysteinemia is often observed in individuals with genetic predispositions, such as methylenetetrahydrofolate reductase polymorphisms, as well as in those with chronic conditions such as CKD. While the direct role of elevated homocysteine in cardiovascular events remains debated, it is considered a modifiable risk factor [22]. The clinical efficacy of lowering homocysteine through dietary changes and B-vitamin supplementation, particularly with folic acid, B6, and B12, for high-risk populations remains debated. Indeed, even if this therapeutic approach has been shown to improve endothelial function and may help reduce cardiovascular risk, particularly in patients with underlying genetic or metabolic susceptibilities, more convincing data are needed [23]. While Lp(a) and hsCRP have a recognized role in cardiovascular risk assessment and guiding treatment strategies in selected patients, routine measurement of other circulating biomarkers as risk modifiers during evaluation is not recommended due to limited evidence [5].

## 3. Cancers and Their Therapies

Cancers and their treatments, including chemotherapy, radiation, and newer therapies such as immune checkpoint inhibitors (ICIs) and tyrosine kinase inhibitors (TKIs), significantly contribute to cardiovascular risk, especially cancers associated with a high thrombotic risk. Commonly used chemotherapeutic agents, like anthracyclines, are linked to dose-dependent cardiomyopathy, while targeted therapies such as trastuzumab can cause reversible left ventricular dysfunction, especially in patients treated for HER2-positive breast cancer [24,25]. Radiation therapy, particularly for thoracic malignancies, further increases cardiovascular risk by promoting coronary artery calcification, fibrosis, and other adverse cardiac effects such as pericarditis and conduction abnormalities [26]. Newer oncology treatments, including ICIs and TKIs, have transformed cancer care but present various cardiovascular risks. ICIs, designed to enhance immune response against tumors, have been associated with immune-mediated myocarditis, pericarditis, and arrhythmias. Meanwhile, TKIs contribute to hypertension, vascular events, and, in some cases, heart failure. Due to these potential complications, it is essential to carefully balance the therapeutic benefits of cancer treatment with the risks of cardiovascular toxicity [26]. Given the overlapping risk factors between cancer and CVD such as aging, smoking, and metabolic dysregulation, cancer itself has emerged as an independent risk factor for cardiovascular events, particularly in survivors. As cancer survival rates improve, the long-term cardiovascular effects of both the malignancy and its treatments have become a critical focus in cardio-oncology, emphasizing the importance of routine cardiovascular monitoring for patients undergoing cancer therapies [27]. To minimize these risks, tailored cardiovascular risk assessment strategies are recommended for oncologic patients. Tools such as coronary artery calcium (CAC) scoring, cardiac MRI, and echocardiography seem useful for detecting subclinical atherosclerosis and monitoring cardiac function over time. For instance, CAC scoring after 40 years old provides a quantitative assessment of coronary plaque burden, which is particularly valuable in patients with a history of thoracic radiation, while cardiac MRI is effective in assessing myocardial fibrosis and inflammation [28,29]. A multidisciplinary approach is crucial to decrease negative outcomes for cancer patients facing cardiovascular risks. The involvement of cardio-oncology teams could lead to the development of personalized surveillance and intervention strategies tailored to individual risk profiles and treatment regimens. Screening and optimization of CVD risk factors is highly recommended during cancer therapy. Regular follow-up and monitoring for late-onset cardiovascular effects are especially relevant in high-risk populations, such as those with a history of radiation or anthracycline exposure, helping to mitigate adverse cardiac events and support long-term survival and quality of life [26].

## 4. Chronic Kidney Disease

CKD is a well-established independent risk factor for CVD. The interplay with CVD is driven by multiple mechanisms including disrupted mineral metabolism, vascular calcification, and chronic inflammation. CKD-related cardiovascular risk is compounded by CKD-specific factors such as the retention of uremic toxins, altered lipid metabolism, and oxidative stress, all of which contribute to accelerated atherogenesis and arterial stiffness, raising the risk of myocardial infarction and heart failure [30]. CKD impacts cardiovascular health through several interrelated mechanisms. Reduced renal function leads to imbalances in calcium phosphate levels, promoting vascular calcification and increasing arterial stiffness. Additionally, CKD is frequently associated with traditional cardiovascular risk factors like hypertension, dyslipidemia, and insulin resistance, which further exacerbate the development of atherosclerosis. Uremic toxins, such as asymmetric dimethylarginine, contribute to endothelial dysfunction, increasing the probability of cardiovascular events [31].

CKD is associated with significant alterations in lipid metabolism, contributing to increased cardiovascular risk. Dyslipidemia observed in CKD includes elevated TG and Lp(a), along with reduced HDL-C. These lipid abnormalities result from impaired catabolism of triglyceride-rich lipoproteins, downregulation of lipoprotein lipase activity, and altered hepatic synthesis of apolipoproteins. The elevation of Lp(a) in CKD is particularly relevant, as impaired renal clearance leads to its accumulation, exacerbating atherosclerosis and valvular calcification. Additionally, HDL-C levels are typically reduced due to enhanced oxidative modifications, decreased reverse cholesterol transport, and impaired antioxidant functions [32,33]. Effective management of cardiovascular risk in CKD patients requires early and accurate risk stratification. Key assessments include estimated glomerular filtration rate (eGFR) and albuminuria, which correlate with adverse CVD outcomes. Indeed, patients with lower eGFR and higher albuminuria levels are at greater risk for heart failure, myocardial infarction, and stroke. Other markers, such as elevated fibroblast growth factor-23 and parathyroid hormone, have also been linked to adverse cardiovascular outcomes, especially in advanced CKD stages [34]. Managing cardiovascular risk in CKD patients involves a multifaceted therapeutic approach targeting both renal and cardiovascular health. Angiotensin-converting enzyme inhibitors (ACE-is) and angiotensin II receptor blockers (ARBs) are key therapies in CKD management, shown to slow disease progression and reduce cardiovascular events by controlling blood pressure and mitigating albuminuria. Sodium–glucose cotransporter-2 inhibitors are also effective in CKD patients, decreasing cardiovascular morbidity and mortality (particularly heart failure-related events), as well as renal protection. Other therapies, such as statins, which are indicated in CKD due to the high cardiovascular risk associated with the disease, and phosphate binders for mitigating vascular calcification, are critical for comprehensive CKD management [35]. By integrating cardiovascular assessments into routine CKD management and implementing evidence-based therapeutic strategies, clinicians can address the renal and cardiovascular complications associated with CKD. This holistic approach not only improves cardiovascular outcomes but also enhances overall survival and quality of life for individuals with this prevalent condition [36].

Other therapies, such as statins, which are indicated in CKD due to the high cardiovascular risk associated with the disease, and phosphate binders, which help mitigate vascular calcification, are critical for comprehensive CKD management

## 5. Chronic Obstructive Pulmonary Disease

COPD is a leading cause of respiratory morbidity and mortality, and it significantly elevates cardiovascular risk due to systemic inflammation and oxidative stress, which contribute to atherosclerosis and vascular dysfunction. Shared risk factors such as smoking, sedentary lifestyle, and metabolic disorders further exacerbate cardiovascular morbidity in COPD patients [37]. Chronic inflammation associated with COPD is characterized by elevated levels of inflammatory markers like hs-CRP, interleukin-6, and tumor necrosis factor-alpha, all of which correlate with increased rates of cardiovascular events, including myocardial infarction, heart failure, and stroke [38]. Furthermore, exacerbations of COPD can destabilize heart failure [39]. Comprehensive cardiovascular risk assessment has a key role in COPD patients due to their elevated risk. Routine evaluations should include blood pressure monitoring, lipid profiles, and biomarkers of inflammation, such as hs-CRP. Imaging techniques such as echocardiography, carotid intima-media thickness measurement, and CAC scoring are indicated for detecting subclinical atherosclerosis [40]. Addressing cardiovascular risk in patients with COPD requires a comprehensive and multidisciplinary strategy. Effective management of COPD exacerbations is crucial, as flare-ups increase the risk of myocardial infarction and arrhythmias. Pharmacologic interventions, including beta-agonists, anticholinergics, and inhaled corticosteroids, alleviate respiratory symptoms but require careful consideration due to potential cardiovascular side effects. Long-acting beta-agonists and anticholinergics can improve outcomes but should be used cautiously in patients with pre-existing cardiovascular conditions. Additionally, therapies targeting systemic inflammation, such as phosphodiesterase-4 inhibitors, can benefit both pulmonary and cardiovascular health [41]. Lifestyle modifications play a critical role in managing COPD and reducing cardiovascular risk. Smoking cessation, regular physical activity, and dietary adjustments are fundamental components of comprehensive care. Pulmonary rehabilitation programs, which combine exercise training, education, and nutritional support, have been shown to improve cardiovascular function and quality of life in COPD patients. For those with comorbid conditions like hypertension or heart failure, the judicious use of medications such as ACE-i, ARBs, and statins can further reduce cardiovascular risk [42].

## 6. Environmental Exposure

Environmental exposures, in particular air pollution, have been increasingly recognized as emerging risk factors for CVD. Fine particulate matter (PM2.5 and PM10), along with pollutants like nitrogen dioxide and ozone, are especially harmful due to their ability to enter the bloodstream, triggering oxidative stress and systemic inflammation that accelerate atherosclerosis. Short-term increases in PM2.5 concentrations have been linked to acute cardiovascular events, such as myocardial infarction and stroke, while long-term exposure is associated with chronic conditions including hypertension, heart failure, and atrial fibrillation [43]. Climate change has intensified the frequency and severity of extreme weather events, such as heatwaves, wildfires, and severe storms, all of which impact cardiovascular health. Heatwaves can lead to dehydration, electrolyte imbalances, and heat stress, putting additional strain on the cardiovascular system. Wildfires not only increase exposure to PM2.5 but also release harmful chemicals that exacerbate respiratory and cardiovascular conditions [44,45]. Mitigating the cardiovascular risks associated with environmental exposure requires a combination of public health measures, patient education, and policy interventions. Community-level efforts, such as regulating industrial emissions and promoting clean energy, are essential in reducing pollutant exposure. On an individual level, patients can be advised to minimize outdoor activities during high pollution days and stay hydrated during heatwaves. Additionally, healthcare providers play a key role in advocating for broader policy measures to address climate change and protect vulnerable populations from its cardiovascular impacts [46]. More recently, it has become progressively clear that water and soil pollution also significantly contribute to cardiovascular risk by introducing harmful substances such as heavy metals, pesticides, and microplastics into the environment and food chain. These pollutants can disrupt metabolic and endocrine pathways, promote systemic inflammation, and exacerbate atherosclerosis through chronic exposure. Additionally, contaminated water sources increase the risk of hypertension and other cardiovascular conditions by delivering toxins directly to human populations. Addressing these environmental challenges is critical for mitigating their impact on public health and reducing the global burden of CVD [47]. Recent experimental findings have demonstrated the presence of microplastics in human cardiovascular structures, including carotid arteries, coronary arteries, and aortas, suggesting their potential role as markers or determinants of adverse cardiovascular events. These microscopic plastic particles, derived from environmental exposure and industrial pollutants, have been detected in vascular tissues where they may contribute to endothelial injury, inflammatory cascades, and atherosclerotic plaque formation. The identification of microplastics in such critical sites raises concerns about their direct involvement in promoting vascular calcification, oxidative stress, and impaired arterial compliance. Their chemical components, often containing endocrine-disrupting additives, could further exacerbate their toxicological impact on vascular health. While the mechanistic pathways linking microplastics to cardiovascular events are still being elucidated, their presence seems to correlate with heightened risks of myocardial infarction, stroke, and other ischemic conditions. These findings emphasize the importance of addressing microplastic exposure as a potential modifiable risk factor in cardiovascular prevention strategies and highlight the need for further research to clarify their pathological role and clinical significance [48,49,50,51].

Furthermore, environmental noise pollution (e.g., road traffic and aircraft) has emerged as an important but often overlooked cardiovascular risk factor. Chronic noise exposure has been linked to increased risks of hypertension, myocardial infarction, and stroke through mechanisms involving autonomic nervous system activation, oxidative stress, endothelial dysfunction, and metabolic disturbances [52]. A case-crossover analysis highlighted a significant short-term association between urban noise levels and cardiovascular mortality, independent of air pollution [53]. Household air pollution, primarily from cooking and heating methods using biomass fuel, coal, and inefficient stoves, is a key factor in indoor air pollution. Exposure to household air pollution has been associated with increased cardiovascular risk through mechanisms such as oxidative stress, systemic inflammation, and endothelial dysfunction [54]. By incorporating environmental risk factors into cardiovascular care and supporting both individual and collective preventive strategies, healthcare professionals can help reduce the CVD burden linked to environmental exposure [43].

## 7. Chronic Inflammatory Diseases

To date, autoimmune diseases’ role in increasing CVD prevalence is widely recognized. Although many pathophysiological mechanisms are yet to be determined, many have been found. Systemic sclerosis, Addison’s disease, systemic lupus erythematosus, and type 1 diabetes are the autoimmune diseases which are associated with the highest cardiovascular risk [55]. The increased cardiovascular risk in inflammatory conditions is primarily driven by chronic low-grade inflammation within the arterial wall, leading to endothelial dysfunction, accelerated atherosclerosis, and heightened thrombogenicity. This process, often referred to as ‘inflammaging’, is particularly relevant in conditions such as rheumatoid arthritis and systemic lupus erythematosus. This mechanism seems to contribute to the increasing cardiovascular death observed in patients with rheumatoid arthritis [55]. Other autoimmune diseases such as ankylosing spondylitis also are associated with an increase in cardiovascular events [56,57], although the risk-profile is different for every single event. Other conditions, such as psoriasis, are associated with increased adverse cardiovascular outcomes, such as death, ischemic heart disease, and arrhythmias. Psoriasis is an independent risk factor of CVD [58] as cardiovascular risk might be related through a cause–effect link [59], i.e., through the increased incidence of atherosclerosis and dyslipidemia in the patients affected by both the conditions [60]. More inflammatory conditions, such as inflammatory bowel diseases (IBDs), are linked to an increase in endothelial dysfunction [61] and atherosclerosis [62], coronary heart disease, and stroke [61,63,64]. In such conditions, several pathways are related to a pro-inflammatory state which eventually activates monocytes, leading to inflammation and tissue damage, eventually becoming a risk factor for CVD. Among patients affected by IBD, those who have Crohn’s disease tend to have higher cardiovascular risk when compared to those who have Ulcerative Colitis [65].

## 8. Infections and Gut Microbiota

Infections are emerging contributors to cardiovascular risk, capable of precipitating acute cardiovascular events and influencing long-term cardiovascular health [66]. A multi-cohort study ran in the UK found an increased cardiovascular risk for major CVD events immediately after hospitalization in patients with infections which were severe enough to require hospital treatment. These infections also lead to an increased long-term risk [66]. Sometimes, the infection can act as a trigger for cardiovascular events, such as influenza virus infections [67,68], while, sometimes, e.g., in pneumonia [69], the link is an increased short- and long-term risk of CVDs. Some infections can be related to CVDs in both ways, by triggering events and by increasing overall cardiovascular risk, such as S. pneumoniae [70]. Other common microorganisms, such as Epstein–Barr virus, are related to rare but severe CV complications [71], while others, such as Human Cytomegalovirus, can contribute to an increased cardiovascular risk in immunodeficient patients through the development of atherosclerosis [72]. Further microorganisms, such as H. pylori, are associated with a mildly increased risk of CVDs [73]. SARS-CoV-2 infection is related to severe endothelial damage and is, therefore, an important and evolving factor of cardiovascular risk, especially in the long-COVID form [74]. HIV infection is related to an increased risk of CVD; such risk persists even after antiviral therapy has begun. Its risk is related to pro-inflammatory effects of HIV proteins, CD4+ depletion, and the increase in atherogenesis [75]. Likewise, periodontal disease is associated with an increased risk of CVDs, especially myocardial infarction. Periodontitis is a chronic inflammatory disease mainly caused by Gram-negative bacteria. Further studies are needed to define the clear role of preventive and therapeutic strategies in reducing CVD risk [5]. SARS-CoV-2 infection has emerged as a significant cardiovascular risk modifier, both acutely and in the long term. Acute COVID-19 can cause myocardial injury, endothelial dysfunction, and a hypercoagulable state, increasing the risk of myocardial infarction, stroke, and venous thromboembolism. Long-COVID has also been associated with persistent cardiac symptoms, including arrhythmias, autonomic dysfunction, and post-inflammatory myocardial fibrosis, further emphasizing the need for cardiovascular monitoring in affected patients [76]. Nonpathological interactions between humans and microorganisms, such as in the gut microbiome, can also be related to cardiovascular issues [77]. Although the connection between a specific change in microbiome composition and a certain cardiovascular issue is yet to be defined, it seems likely that the abundance in gut microbiota of STEMI patients of species like Colinsella stercoris, Flavonifractor plautii, and Ruthenibacterium lactaiformans might be related with inflammation and lipid metabolism pathways, which are eventually key factors in CAD development [78]. Furthermore, other factors potentially involved in the cardiac outcome affection of gut microbiota are endotoxemia and immunological dysfunction [79], and some other studies found a potential correlation between gut microbiota, dysbiosis, and chronic heart failure [80].

## 9. Sleep Disorders and Cardiovascular Risk

Sleep disorders are connected to cardiovascular health through several pathways, including inflammation, autonomic nervous system disruption, endothelial dysfunction, and altered metabolic processes. These disorders cause higher heart sympathetic tone and, therefore, a sympathetic overactivity, eventually related to nocturnal oscillation in arterial pressure and hypertension. The importance of such an issue regarding cardiovascular risk is related to the available interventions which can mitigate this impact, like lifestyle changes, behavioral therapies, and positive airway pressure (PAP) [81]. Central sleep apnea is recognized as a major risk factor for atrial fibrillation (AF) and, therefore, for many cardiovascular issues [82]. Although being the most studied and widely accepted cardiovascular-related risk factor, many other studies over sleep characteristics and their relationship with cardiovascular risk revealed that snoring, daytime sleepiness, altered sleep duration, and the mixture of these are considered high-risk sleep patterns. Patients aged over 40 having such characteristics tend to have a higher cardiovascular risk, especially in case of association with blood pressure and diabetes [83]. Younger patients can also be exposed to bad sleep health during their youth, with this becoming a risk factor for future CVD, especially in the case of OSA [84]. OSA is also associated with an increased risk of recurrent MACEs in patients with ACS [85]. In addition to OSA, other sleep disorders, including insomnia, circadian rhythm disturbances, and chronic sleep deprivation, have been linked to increased cardiovascular risk [86]. Poor sleep quality can contribute to obesity, insulin resistance, and hypertension, exacerbating cardiometabolic risk. Shift workers, in particular, exhibit increased cardiovascular morbidity due to altered circadian regulation and metabolic dysregulation associated with irregular eating patterns and sleep deprivation [87].

## 10. Sex-Specific Conditions

The health impact of traditional cardiovascular risk factors varies by gender, and it has been outlined that they are associated with higher hazard ratios for some ACS phenotypes in women than in men [88]. Therefore, the importance of integrating sex, gender, and even gender identity considerations into the risk assessment and patient clinical management has been recognized by the current guidelines [5]. It is well known that inflammation and the resulting fibrosis play a pivotal role in the pathogenesis of CVD, and premenopausal women are protected by estrogens which produce anti-inflammatory actions on endothelial and immune cells potentially leading to less maladaptive left ventricular remodeling and, eventually, improved survival [89,90]. In addition, a large body of evidence has shown that sex-specific conditions are important factors as well in determining clinical outcomes. For instance, pregnancy-related disorders significantly affect the risk of future cardiovascular events, meaning that targeted interventions should be initiated soon after delivery. Indeed, hypertensive disorders in pregnancy are relatively common conditions, including gestational hypertension and pre-eclampsia, and it has been pointed out that they may lead to a higher incidence of long-term cardiovascular adverse events, such as myocardial infarction, heart failure, stroke, and cardiovascular death [91,92]. Also, attention should be given to the diagnosis of gestational diabetes mellitus (GDM), because it confers a twofold higher risk of cardiovascular events postpartum, and this risk seems to be independent of the intercurrent development of type 2 diabetes [93]. Another common disorder that should not be overlooked is polycystic ovary syndrome (PCOS): women with PCOS have an increased prevalence of cardiovascular risk factors, mostly due to insulin resistance and subclinical CVD markers such as coronary artery calcium score, C-reactive protein, carotid intima-media thickness, and endothelial dysfunction, but it is not entirely clear whether PCOS is an independent predictor of clinical CVD events [94]. Lastly, Takotsubo syndrome (TTS) is significantly more prevalent in women, particularly postmenopausal women, partly as a consequence of a higher cardiac sympathetic stimulation which results in an imbalance in neuronal norepinephrine homeostasis [95]. Understanding TTS as a sex-specific risk factor arising in most of the cases from intense psychoemotional stress in women emphasizes the importance of tailored approaches in both the diagnosis and management of this condition. Moving to male-related disorders, it has been shown that erectile dysfunction (ED) could be an early marker for latent ischemic heart disease, as the smaller diameter of the penile arteries makes them susceptible to endothelial dysfunction and atherosclerotic changes earlier than larger coronary arteries [96]. Moreover, recent evidence highlighted that ED could be regarded to as a predictor of future CV risk [97], especially in the setting of chronic coronary syndromes (CCSs), possibly as a consequence of an interaction with traditional CV risk factors [98,99]. The use of anabolic androgenic steroids (AASs), both in athletes and non-athletic individuals for aesthetic purposes, has been associated with a heightened risk of cardiovascular disease [100]. Chronic exposure to AASs can lead to endothelial dysfunction, left ventricular hypertrophy, increased blood pressure, and heightened thrombotic risk [101]. Clinicians should be aware of indirect markers of concealed AAS use, including polycythemia, low HDL-C, and signs of left ventricular dysfunction.

## 11. Mental Disorders

Mental disorders, such as anxiety disorders, somatoform disorders, substance use disorders, personality disorders, mood disorders, and psychotic disorders, may increase the risk of CVD and reduce life expectancy in both sexes [102]. In particular, depression, anxiety (i.e., panic disorder, specific phobias, and post-traumatic stress disorder) and alcohol use disorders seem to be significantly associated with heart disease onset, meaning that a careful surveillance for such often-hidden symptoms is crucial [103]. The mechanisms by which mental disorders induce CVD are complex. The mechanisms might depend on socioeconomic factors and the side effects of medications [104] or on the impaired capacity of these patients to refer to healthcare systems or to be adherent to therapies [105] or even on the abuse of psychostimulants which can elicit accelerated atherosclerosis, hypertension, and myocardial ischemia [106]. Moreover, some complex biological pathways between psychosocial stress and CVD have been clarified: chronic stress conditions can increase the resting metabolic activity in the amygdala, a key neural center involved in the emotional and physiological response to stress, leading to increased bone marrow activity (an index of leukopoiesis) and arterial wall inflammation, ultimately resulting in an increased risk of adverse cardiovascular events [107]. Furthermore, higher baseline metabolic activity in the amygdala was found to be associated with higher insulin resistance, which might augment the risk of new subsequent diabetes [108]. Finally, a bidirectional association between mental disorders and CVD has been described, advocating an increased vigilance for individuals with these co-morbid conditions [109].

## 12. Psychosocial Factors

In recent years, greater attention has been devoted to psychosocial factors, especially acute and chronic stressors such as childhood trauma, work stress, and social isolation, since they can promote atherosclerotic CVD development and progression independently of conventional risk factors [110]. The pathophysiological process that has been proposed to underly these adverse effects is the dysregulation of the hypothalamic–pituitary–adrenal (HPA) axis, which results in a higher release of glucocorticoids, including cortisol, into the blood stream [111]. In turn, chronic cortisol excess affects plasma lipoprotein metabolism causing hyperlipidemia, activates liver gluconeogenesis contributing to insulin resistance, and favors mineralocorticoid-induced sodium retention inducing hypertension [112]. Thus, there is a strong body of evidence arguing that conditions of corticosteroid excess, either endogenous or exogenous, clearly result in an excess of cardiovascular events, especially in an increased risk of heart failure rather than ischemic heart disease [113]. Compared to its previous version, the American Heart Association’s Life’s Essential 8 emphasizes the importance of psychological and behavioral factors in the primordial prevention of CVD, collectively referred to as the mind–heart–body connection. This paradigm shift underscores the significant interplay between mental health, stress management, and cardiovascular well-being [114]. Evidence shows that negative psychological states, such as chronic stress, depression, and anxiety, not only heighten cardiovascular risk but do so by exacerbating inflammation, impairing endothelial function, and fostering unhealthy behaviors like smoking and poor dietary habits. On the other hand, positive psychological attributes, including optimism and resilience, are associated with better cardiovascular outcomes and improved adherence to preventive health behaviors [115]. Integrating these elements into cardiovascular health highlights a holistic approach that goes beyond traditional metrics, addressing psychosocial determinants of health. This paradigm shift focuses on tackling the root causes of cardiovascular risks through a comprehensive, life-course framework, emphasizing that mental health is as vital as physical health in achieving cardiovascular longevity.

## 13. Migraine with Aura

Another cardiovascular risk factor which has progressively gained more and more attention is migraine. Migraine is a common condition as it is estimated to affect around 15% of the population, and migraine with aura accounts for one-third of all migraines [5,116]. Migraine has been associated with an increased long-term risk of stroke (both ischemic and hemorrhagic) and myocardial infarction, with this association being stronger in the forms with aura [117]. Nonetheless, the pathophysiological link between migraine with aura and CVD remains elusive: it has been supposed that the presumed substrate of aura, a self-propagating wave of neuronal and glial sustained depolarization moving through intact brain tissue, called cortical spreading depression (CSD), may predispose cerebral ischemic events by reducing cerebral blood flow and by upregulating pro-inflammatory mediators [118]. However, evidence is less consistent regarding the relationship with heart disease, although it has been hypothesized that the neuropeptide release and the autonomic activation induced by migraine attacks could unmask coronary artery disease or contribute to the development of vasculopathy over the course of multiple episodes [119]. Accordingly, this relationship may be better explained considering shared risk factors and comorbidities.

## 14. Discussion

**Figure 2:**
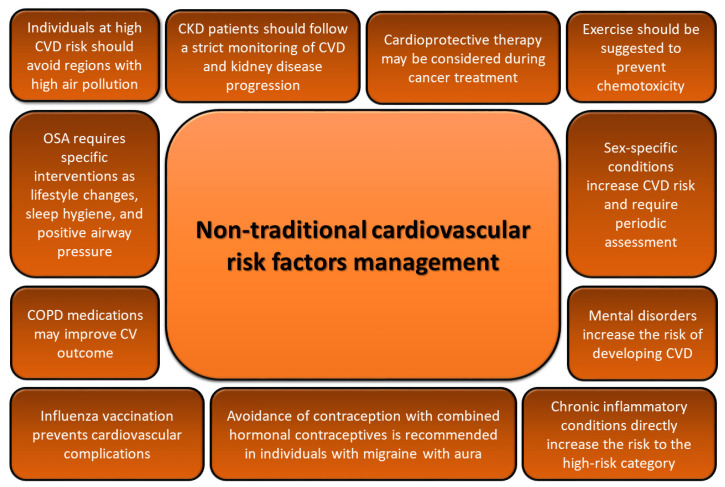
Tailored management of patients with non-traditional cardiovascular risk factors. COPD, chronic obstructive pulmonary disease; CV, cardiovascular; CKD, chronic kidney disease; CVD, cardiovascular disease; OSA, obstructive sleep apnea.

Non-traditional cardiovascular risk factors should be considered during patient assessment and management, from first presentation to long-term follow-up. In clinical practice, cardiovascular risk is assessed using risk charts (e.g., SCORE2 and SCORE2-OP) that consider conventional and traditional risk factors to estimate 10-year CVD risk. However, as non-traditional risk factors play a key role in modifying the calculated risk and the management and clinical impact of patients, their assessment can impact the estimation of the risk by changing its category. Indeed, in patients at moderate CVD risk, non-traditional risk factors can modify the calculated risk with a significant impact. In this category of patients, their role is relevant in modifying the risk close to the decision threshold. The reclassification of cardiovascular risk from moderate to high totally changes the management and treatment of patients and, above all, the prognosis. Cardiovascular imaging may help to better define the risk, but the cost-effectiveness of this modern approach remains an issue. Therefore, further studies are needed to define the list of potential non-traditional risk modifiers and their subsequent impact on CVD outcome. Emerging studies are considering the use of genetics in prevention, with polygenic risk scores (PRSs) for risk assessment and improvement of atherosclerotic CVD prediction. However, as with biomarkers, the routine use of genetic risk scores is not recommended [5]. Furthermore, behavioral habits, including eating patterns, could be considered non-traditional cardiovascular risk factors due to their significant influence on metabolic health, inflammation, and other processes associated with CVD. Therefore, several assessments should be performed such as assessing dietary patterns and meal timing during clinical evaluations, monitoring eating speed and emotional eating habits, and promoting education on portion control and the benefits of dietary patterns like the Mediterranean diet. Among non-traditional risk factors, psychosocial stressors are the main factors indicated as risk modifiers, and careful assessment for this condition is suggested. Indeed, a previous study documented a positive impact of routine depression screening on long-term atherosclerotic CVD outcomes [120]. After establishing the patient’s cardiovascular risk considering both traditional and non-traditional risk factors, treatment decisions should be performed following a tailored approach. Indeed, in addition to treating and achieving well-established targets for conventional risk factors, non-traditional risk factors should also be treated at the individual and population levels. At the individual level, non-traditional risk factor assessment allows for the targeted identification of high-risk patients, enabling tailored interventions (Figure 2); specifically, individuals at high CVD risk should try to avoid regions with high air pollution; CKD patients should follow strict monitoring for CVD and kidney disease progression; cardioprotective therapy may be considered during cancer treatment to avoid reduction in left ventricle systolic function, and exercise should also be suggested to prevent chemotoxicity; COPD medications (e.g., long-acting muscarinic antagonists and long-acting beta agonists) may improve cardiovascular outcomes and are not associated with adverse events in stable patients, although they require constant monitoring and attention in patients with pre-existing cardiovascular conditions; chronic inflammatory conditions directly increase the risk to the high-risk category, which varies with the level of disease activity, and optimal cardiovascular and anti-inflammatory treatment is recommended; influenza vaccination prevents cardiovascular complications of this infection; smoking cessation and avoidance of contraception with combined hormonal contraceptives are recommended to reduce cardiovascular risk in individuals with migraine with aura; OSA is the most important sleep disturbance with a strong CVD association that requires specific interventions such as lifestyle changes, sleep hygiene, and PAP, if needed; mental disorders increase the risk of developing CVD and their presence should be assessed cautiously; sex-specific conditions, such as pre-eclampsia, pregnancy-induced hypertension, polycystic ovary syndrome, and erectile dysfunction, increase CVD risk and require periodic assessment. At the population level, interventions informed by non-traditional risk factors may benefit individuals with borderline or “marginally normal” states. For instance, policies to lower sodium content in processed foods target not only hypertensive individuals but also those with prehypertension or normotension, reducing population-level blood pressure and preventing future cardiovascular events [121]. Also, broader initiatives addressing psychosocial stress, such as workplace wellness programs or community-based interventions, can reduce cardiovascular risk in populations exposed to chronic stress [122].

Integrating non-traditional cardiovascular risk factors into routine practice poses both opportunities and challenges for diverse healthcare systems. These offer a nuanced view of cardiovascular risk beyond traditional metrics. However, their incorporation into routine risk assessments is hindered by limited standardization, variability in diagnostic tools, and lack of integration into established risk charts. Furthermore, disparities in healthcare infrastructure across regions complicate the adoption of advanced diagnostics and personalized care strategies. To address these challenges, healthcare systems must invest in education, streamline biomarker testing, and promote interdisciplinary collaboration to ensure equitable access to comprehensive risk assessment tools. Despite these hurdles, tailored approaches leveraging non-traditional risk factors hold the potential to improve risk stratification and ultimately reduce CVD burden globally. Future research directions should follow two pillars. First, further studies are needed to define the non-traditional risk factors to be included in CVD risk charts. Second, a clear list of potential risk modifiers to consider in management decisions and risk reclassification should be established. Furthermore, as risk modifiers, non-traditional CVD risk factors should also demonstrate their public health benefit, positive impact on cardiovascular outcome, and feasibility in daily clinical practice. Traditional risk factors represent a part of total CVD burden and considering them as the sole cause of CVD risk could still leave the patient at risk of future adverse events. Thus, non-traditional risk factors play an important role in risk assessment and reclassification, and their appropriate management can reduce total CVD burden. Therefore, the high residual risk that some patients have despite treatment of traditional risk factors could be explained by this inaccurate assessment of total CVD risk. New risk scores combining traditional and non-traditional cardiovascular risk factors are needed to better assess the patient, minimize risk, and change adverse cardiovascular outcome.

## 15. Conclusions

Non-traditional cardiovascular risk factors influence cardiovascular risk assessment and require a specific approach and management. Careful evaluation of these factors should be performed during initial CVD risk assessment and during follow-up. Indeed, as they may impact on the prognosis, efforts are required to better define the cardiovascular risk. Patients with non-traditional risk factors should benefit from specific and tailored management based on their individual cardiovascular profile. Furthermore, treatments should be suggested at individual and population levels to reduce CVD burden. Non-traditional cardiovascular risk factors represent a major challenge to reducing the residual and total risk of further MACEs and mortality.

## Figures and Tables

**Table 1:** Key non-traditional cardiovascular risk factors.

Risk Factor	Definition	Prevalence	Features	Prognostic Impact	Strategies for Management	Clinical Example
Chronic Kidney Disease	Condition characterized by progressive loss of kidney function.	Affects ~10% of adults globally.	Retention of uremic toxins, vascular calcification, chronic inflammation, and the presence of atherogenic lipoproteins.	Accelerates atherosclerosis and arterial stiffness and increases risk of heart failure and MI.	Control BP with ACE inhibitors/ARBs; manage lipids with statins; utilize SGLT2 inhibitors for renal and cardiac protection.	A 63-year-old man with CKD (eGFR < 45 mL/min/1.73 m^2^) presenting with MI.
COPD	Chronic inflammatory lung disease that causes obstructed airflow.	Affects 5–10% of adults worldwide.	Systemic inflammation, oxidative stress, and shared risk factors like smoking.	Increases risk of MI, stroke, and arrhythmias.	Manage exacerbations, use inhaled medications cautiously, and incorporate pulmonary rehabilitation to improve outcomes.	A 68-year-old woman with COPD exacerbations and atrial fibrillation.
Environmental Exposure	Exposure to pollutants like PM2.5, nitrogen dioxide, and microplastics.	Widespread; varies by geographic location and industrial activity.	Triggers oxidative stress, systemic inflammation, and endothelial dysfunction.	Linked to acute events like MI and stroke; chronic exposure increases risk of hypertension and heart failure.	Minimize outdoor activities during high pollution; advocate for clean energy policies; promote individual protective measures (e.g., masks and air purifiers).	A 45-year-old man exposed to heavy air pollution with elevated BP and plaque build-up on imaging.
Inflammatory Conditions	Autoimmune diseases like RA and SLE.	1–2% of the population affected by RA; SLE affects 0.1%.	Chronic inflammation, immune dysregulation, and endothelial dysfunction.	Significantly increases cardiovascular mortality, including MI and stroke.	Optimize treatment of inflammation with disease-modifying antirheumatic drugs (DMARDs); regular cardiovascular monitoring and risk reduction strategies.	A 55-year-old woman with RA and recurrent chest pain diagnosed with coronary artery disease.
Lipoprotein(a)	Genetically determined lipoprotein variant associated with atherogenic and pro-thrombotic properties.	Elevated in 20–30% of the general population globally.	Promotes foam cell formation, oxidative LDL modification, and endothelial dysfunction.	Increases risk of MI, stroke, peripheral artery disease, and aortic stenosis.	Measure levels at least once; consider PCSK9 inhibitors or antisense therapies in high-risk patients.	A 58-year-old woman with premature MI and a family history of CAD showing elevated Lp(a).
Psychosocial Stress	Chronic or acute stressors impacting hypothalamic–pituitary–adrenal axis regulation.	Highly variable; common in lower socioeconomic groups.	Dysregulated cortisol levels, hyperlipidemia, and insulin resistance.	Elevated risk of heart failure, ischemic heart disease, and stroke.	Cognitive–behavioral therapy, stress management, and lifestyle modifications; consider treating comorbid mental health disorders.	A 40-year-old woman with a stressful job, insomnia, and new-onset hypertension.
Sleep Apnea (OSA)	Sleep disorder causing repetitive upper airway obstruction during sleep.	10–20% of adults (higher in obese individuals).	Increased sympathetic tone, nocturnal BP spikes, and hypoxia.	Strongly linked to hypertension, atrial fibrillation, and recurrent major adverse cardiovascular events.	Lifestyle changes, weight loss, and continuous positive airway pressure therapy.	A 50-year-old obese man with daytime fatigue and poorly controlled hypertension.
hsCRP	Biomarker of systemic inflammation correlated with atherosclerosis and major adverse cardiovascular events (MACEs).	Elevated in many patients with CVD, especially those with metabolic syndrome.	Endothelial dysfunction, plaque instability, and increased thrombosis risk.	Associated with higher risk of myocardial infarction, stroke, and cardiovascular death.	Lifestyle modifications; statins and anti-inflammatory agents (e.g., colchicine) can lower hsCRP.	A 55-year-old with metabolic syndrome and hsCRP >2 mg/L at risk for future MI.
Noise Pollution	Chronic exposure to high-intensity sound levels (e.g., traffic and industrial noise) leading to physiological stress responses.	Urban areas, industrial zones, and high-traffic regions are most affected.	Increased stress hormone levels, endothelial dysfunction, and autonomic nervous system activation.	Higher risk of hypertension, atrial fibrillation, and cardiovascular events.	Soundproofing, noise regulations, public policy interventions, and personal protection measures.	A 48-year-old living near an airport with new-onset hypertension and stress-related arrhythmias.

ACE, angiotensin-converting enzyme; ARB, angiotensin receptor blocker; BP, blood pressure; CAD, coronary artery disease; CKD, chronic kidney disease; COPD, chronic obstructive pulmonary disease; eGFR, estimated glomerular filtration rate; hsCRP, high-sensitivity C-reactive protein; LDL, low-density lipoprotein; Lp(a), lipoprotein(a); MI, myocardial infarction; PM, particulate matter; PCSK9, proprotein convertase subtilisin/kexin type 9; RA, rheumatoid arthritis; SGLT2, sodium–glucose cotransporter 2; SLE, systemic lupus erythematosus.

## Data Availability

No new data were created or analyzed in this study. Data sharing is not applicable to this article.
